# Correction: Antibacterial drug discovery: challenges and preclinical promises from synthetic small molecules

**DOI:** 10.1039/d6cs90068b

**Published:** 2026-08-07

**Authors:** Guilherme F. S. Fernandes, Seong-Heun Kim, Jean L. Dos Santos, Yuhang Wang, Christelle Soudy, Joanna Redmond, Daniele Castagnolo, Matthew Todd

**Affiliations:** a Chemical Biology Science Technology Platform, The Francis Crick Institute London NW1 1AT UK guilherme.fernandes@crick.ac.uk christelle.soudy@crick.ac.uk joanna.redmond@crick.ac.uk; b School of Pharmacy, University College London London WC1N 1AX UK matthew.todd@ucl.ac.uk yuhang.wang.19@ucl.ac.uk; c Institute of Pharmaceutical Science, King’s College London London SE1 9NH UK seong_heun.2.kim@kcl.ac.uk; d School of Pharmaceutical Sciences, São Paulo State University Araraquara 14800903 Brazil jean.santos@unesp.br; e Department of Chemistry, University College London London E20 2AE UK d.castagnolo@ucl.ac.uk

## Abstract

Correction for ‘Antibacterial drug discovery: challenges and preclinical promises from synthetic small molecules’ by Guilherme F. S. Fernandes *et al.*, *Chem. Soc. Rev.*, 2026, **55**, 7853–7932, https://doi.org/10.1039/d5cs00617a

The authors regret that a reference in the original article was omitted in error. The missing reference should have been cited at the end of the second paragraph in section 4 on page 7859. The reference is cited here as ref. 1.

The Royal Society of Chemistry apologises for these errors and any consequent inconvenience to authors and readers.
